# How intelligence and emotional control are related to suicidal behavior across the life course *– A register-based study with 38-year follow-up*

**DOI:** 10.1017/S0033291719002423

**Published:** 2020-10

**Authors:** Nora Hansson Bittár, Daniel Falkstedt, Alma Sörberg Wallin

**Affiliations:** 1 Department of Clinical Neuroscience, Karolinska Institutet, Stockholm, Sweden; 2 Department of Public Health Sciences, Karolinska Institutet, Stockholm, Sweden

**Keywords:** Emotional control, intelligence, life course, neuroticism, suicide

## Abstract

**Background:**

Both low intelligence and low emotional control have previously been linked to a higher risk of suicide, but it is unknown whether the associations apply consistently over the life course.

**Methods:**

The study was based on data on intelligence and emotional control, collected from 48 738 Swedish men conscripted in 1969–1970, at ages 18–20 years. The data were linked to national registers giving information on subsequent suicidal behavior (completed and attempted suicide) up to the age of 59 years. The associations were investigated using logistic regression and Cox proportional hazards regression models, with adjustment for childhood socioeconomic status.

**Results:**

Intelligence and emotional control assessed in late adolescence both showed robust inverse associations with suicidal behavior over the 38-year follow-up. However, while the association between lower intelligence and higher rate of suicidal behavior remained the same throughout (~40% increased hazard per unit on a five-level scale), the association between lower emotional control and suicidal behavior was substantially stronger in early adulthood (~100% increased hazard per unit) than in late middle age (~30% increased hazard per unit).

**Conclusions:**

The study adds to previous research by showing that the association between poor emotional control and subsequent suicide risk in men becomes weaker over the life course, while the association between low intelligence and suicide risk seems to be constant. The particularly high suicide risk of young men with poor emotional control may motivate targeted prevention efforts.

## Introduction

Suicide is one of the leading causes of death worldwide, and approximately one million people die each year from suicide (World Health Organization, 2012). Like many other health-related outcomes, suicide is not the consequence of a single disease factor. Rather, there are multiple factors that contribute to completed and attempted suicide (here denoted as suicidal behavior), such as the presence of psychiatric disorders, physical illnesses, impulsive behaviors, and life stressors (Gunnell and Lewis, 2005). Previously, it has been shown that both lower level of intelligence and higher level of neuroticism, or low emotional control, are related to an increased risk of suicidal behavior (Allebeck *et al*., 1988; Brezo *et al*., 2006; Batty *et al*., 2010; Gravseth *et al*., 2010; Sörberg *et al*., 2013). However, little is yet known about these associations from a life-course perspective, with regards to whether the associations change or remain stable over time.

Intelligence is a general ability that reflects a broad and deep capability of understanding our surroundings, which involves the ability to reason, plan, solve problems, think abstractly, comprehend complex ideas, learn quickly, and learn from experience (Gottfredson, 1997). Research in recent years has provided extensive evidence that intelligence is an important predictor of major life outcomes, such as educational attainment, occupation, and health, and also suicidal behavior (Batty *et al*., 2010; Sörberg *et al*., 2013; Plomin and Deary, 2015). Moreover, intelligence is fairly stable over the life course. For example, in a Scottish cohort of about 1000 women and men, the raw stability coefficient for people who completed the same intelligence test at age 11 and age 70 was on average 0.67 (Gow *et al*., 2011).

Emotional control is a construct aimed to capture factors such as susceptibility to stress, anxiety, psychosomatic symptoms, and poor control over nervousness and aggression (Potijk *et al*., 2016). This construct has not been widely used in research on suicidal behavior but shows great similarities with one of the big five personality dimensions, namely neuroticism, which is associated with anxiety, anger, depression, impulsiveness and vulnerability (Chamorro-Premuzic, 2016). Although emotional control and neuroticism are not identical constructs, they are highly overlapping. Both concern stable personality traits related to emotional stability, and both have been shown to be associated with an increased risk of suicidal behavior (Allebeck *et al*., 1988; Brezo *et al*., 2006; Wiktorsson *et al*., 2013). The societal economic burden attributed to neuroticism exceeds that of common mental disorders (Cuijpers *et al*., 2010).

Neuroticism has been found to be mostly stable over the life course, but, according to a meta-analysis, a decline in the population mean of neuroticism takes place during late adolescence and early adulthood (Roberts *et al*., 2006). After the age of 30, however, the level of neuroticism tends to regain stability at the group level (Roberts *et al*., 2006). Specifically, test-retest consistency increases with age from 0.45 during adolescence to 0.60 during young and middle adulthood (Roberts and DelVecchio, 2000).

Even though both lower intelligence and higher neuroticism have previously been linked to an increased risk of subsequent suicidal behavior (Allebeck *et al*., 1988; Brezo *et al*., 2006; Batty *et al*., 2010; Gravseth *et al*., 2010; Sörberg *et al*., 2013), little is yet known about those associations from a life-course perspective. Risk factors for suicide might act differently over the life course (Gunnell and Lewis, 2005) and, likewise, the roles of intelligence and emotional control might change over time. Indeed, we have found previously that the association between intelligence measured at age 18–20 and subsequent depression was particularly strong in young adulthood, but then attenuated considerably towards late middle age (Lager *et al*., 2017). Previous researchers have stressed that knowledge of risk factors is essential for the effective prevention of suicidal behavior (Gunnell and Lewis, 2005; Nock *et al*., 2008). From that perspective, understanding of the long-term associations between individual differences and suicidal behavior should be of greatest interest.

### Aim

The aim of this study was to investigate how the associations between intelligence and emotional control in early adulthood and subsequent suicidal behavior develop over time. We examined those associations in a Swedish male cohort with measurements from military conscription in 1969–1970. The cohort information was linked to national register data on suicidal behavior, with childhood socioeconomic status (SES) treated as a potential confounder, and followed over 38 years, up to the age of 59.

## Method

### Study population

The study was based on data collected at compulsory conscription for Swedish military service in 1969 and 1970. About 97–98% of all Swedish men were conscripted at that time; the remaining 2–3% were in most cases exempted due to severe disability or congenital disorders. The cohort consisted of 49 321 men born 1949–1951 (aged 18–20). Approximately 2% of all the men who were conscripted during those years were born prior to 1949, and were excluded from the data set in order to obtain as homogeneous a cohort as possible. All information was anonymized before it was made available for research to protect the integrity of the individuals. The research has been approved by the ethical committee at Karolinska Institutet (numbers 2004/5:9–639/5 and 2010/604:32).

From the original cohort, 583 men were excluded due to missing data on intelligence (*n* = 75) and/or emotional control (*n* = 396), and/or death or emigration prior to the start of follow-up (*n* = 175), which left 48 738 men (98.8%) for analysis.

### Material and procedure

#### Conscription in Sweden

The Swedish conscription process included physical and medical assessments, a test of intelligence, an interview with a psychologist, and questionnaires that covered social background, psychological and psychosomatic problems, and adjustment and substance use (Allebeck *et al*., 1988).

#### Intelligence

Intelligence was measured at conscription through a multidimensional test, which included four subtests examining verbal, logic-inductive and visuospatial ability, and technical comprehension. The outcomes were converted into a global score with a normal (Gaussian) distribution on a nine-level scale. The test battery was designed to measure general intelligence, and used primarily to find a position with appropriate learning demands and predict the conscript's ability to profit from education within the military (Ross, 1988). A detailed description of the test is presented elsewhere (Karlsson *et al*., 2010; Sörberg Wallin, 2015). Test-retest reliability was 0.81 for the global score of a group of 107 men retested after one to three years (Ross, 1988). In the current study, the nine-level scale was converted into a five-level scale in order to match the scale of emotional control. Following conversion, the scale approximately corresponded to IQ bands of <82, 82–95, 96–110, 111–126, and >126.

#### Emotional control

Assessment of emotional control was made through a semi-structured interview administered by a trained psychologist. The interview took about 20–30 min, and a summary of the interview process is provided elsewhere (Potijk *et al*., 2016; Ross, 1988). The overall aim of the interview was to measure the conscript's suitability for military service, including tolerance of mental stress. Emotional control was measured as situation-specific regulation of emotions. The psychologist asked the conscripts how they emotionally responded to important events and situations experienced in childhood and adolescence, for example personal conflicts or stress at school or work. Based on the responses, the psychologists rated emotional control on a scale ranging from one (very low) to five (very high). Higher scores on emotional control were allocated to conscripts with high stress tolerance, low anxiety and control over nervousness and aggression, whereas lower scores were allocated to subjects with psychosomatic symptoms, low levels of stress tolerance, anxiety and problems in controlling nervousness and aggression (Stenbacka and Jokinen, 2015). The inter-rater reliability of the psychologists' ratings has been found to be as high as 0.85 (Lilieblad and Ståhlberg, 1977).

#### Suicidal behavior

The conscription data were linked to register data on suicidal behavior, which encompasses both completed and attempted suicide. Information about completed suicides during the period 1973–2008 was collected from the National Cause of Death Register, and information about suicide attempts for the same time period was obtained from the National Hospital Discharge Register. The variables were classified following the International Classification of Disease (ICD) codes for suicide and suicide attempt (ICD-8/9: E950-9; ICD-10: X60-X84), and for events of undetermined intent (ICD-8/9: E980-9; ICD-10: Y10-Y34). The latter was included since previous research has shown that most deaths from events of undetermined intent are likely to be suicides (Jiang *et al*., 2014).

#### Socioeconomic status

Information on childhood SES was collected from the national census in 1960. The SES categories were based on the occupation of the father, if available, but otherwise the mother. The six categories were: (1) unskilled workers, (2) skilled workers, (3) assistant non-manual employees, (4) non-manual employees at intermediate or higher level, (5) farmers, and (6) others or those with no occupation reported.

### Statistical analysis

The associations between intelligence and emotional control at conscription and subsequent suicidal behavior were first estimated for the full follow-up period using Cox proportional hazard models, yielding hazard ratios (HRs) with 95% confidence intervals (CIs). The conscripts were followed until emigration, death, or the first event of suicidal behavior, or until the end of 2008, whichever came first.

The follow-up period was then divided into four approximate decades (1973–1979, 1980–1989, 1990–1999, and 2000–2008). Thereafter, logistic regressions yielding odds ratios (ORs) were used to investigate the associations of intelligence and emotional control with suicidal behavior for each decade separately in the men who were alive and had not emigrated at the start of each period. Tests of interaction with time were performed to test formally whether the associations remained stable over time. The independent variables, intelligence and emotional control, were modeled as continuous variables, with HRs and ORs given for each step decrease on the scales of intelligence and emotional control, and also as categorical variables to obtain HRs and ORs for each level, with the highest levels as the reference categories. All analyses were adjusted for childhood SES. We then repeated the analyses with mutual adjustment for intelligence and emotional control to separate their associations with suicidal behavior from each other.

Additionally, we performed sensitivity analyses by excluding completed suicide from the dependent variable, and also by excluding events with undetermined intent. These analyses were performed to check whether the results were robust to any differences between attempted and completed suicide and between undetermined and certain suicidal intent. All analyses were performed using SAS 9.4 (SAS Institute, Inc., Cary, NC, USA 2012).

## Results

During the total follow-up period (1973–2008), 1725 of the men (3.5%) had a suicidal-behavior event (493 completed and 1232 attempted suicides as their first event). The numbers of men with a suicidal-behavior event across the levels of intelligence and emotional control are shown in Table 1.

**Table 1.** Associations of intelligence (IQ) and emotional control (EC) in 1969/1970 with suicidal behavior 1973–2008

	*N*	Events	(%)	HR^†^/OR^§^	95% CI
(a) IQ, units^a^					
1	4519	326	(7.21)	6.29^**†**^	4.48–8.83
2	11 695	552	(4.72)	4.02^**†**^	2.89–5.60
3	17 058	552	(3.24)	2.72^**†**^	1.96–3.79
4	12 292	257	(2.09)	1.75^**†**^	1.24–2.46
5	3174	38	(1.20)	1.00^**†**^	–
(b) IQ, per unit decrease					
1973–2008	48 738	1725	(3.54)	1.54^**†**^	1.46–1.61
1973–1979	48 738	416	–	1.65^**§**^	1.49–1–82
1980–1989	48 401	619	–	1.57^**§**^	1.45–1.70
1990–1999	47 907	525	–	1.56^**§**^	1.43–1.70
2000–2008	46 728	408	–	1.53^**§**^	1.39–1.69
(c) EC, units^a^					
1	3070	290	(9.45)	6.64^**†**^	4.75–9.28
2	11 786	598	(5.07)	3.46^**†**^	2.50–4.78
3	19 579	555	(2.83)	1.92^**†**^	1.38–2.65
4	11 598	243	(2.10)	1.43^**†**^	1.02–2.01
5	2705	39	(1.44)	1.00^**†**^	–
(d) EC, per unit decrease					
1973–2008	48 738	1725	(3.54)	1.68^**†**^	1.60–1.76
1973–1979	48 738	416	–	2.17^**§**^	1.95–2.40
1980–1989	48 401	619	–	1.84^**§**^	1.69–2.00
1990–1999	47 907	525	–	1.58^**§**^	1.45–1.73
2000–2008	46 728	408	–	1.44^**§**^	1.30–1.59

Parts a and c: Hazard ratios (HRs; marked with ‘**^†^’**) with 95% confidence intervals (CIs) for the entire follow-up period, modelling IQ and EC as categorical variables. Parts b and d: HRs for the entire follow-up and odds ratios (ORs; marked with ‘^**§**^’) for the separate time periods, with 95% CIs, modelling IQ and EC as continuous variables. All models are adjusted for childhood SES.

a One (1) represents the lowest level and five (5) the highest level of intelligence and emotional control. Level 5 is the reference category in these analyses (HR = 1)

The associations of intelligence and emotional control with suicidal behavior followed dose-response patterns, as shown in Table 1 (parts a and c, IQ and EC in units, modelled as categorical variables). For example, men in the lowest intelligence category (1) had a six-fold higher risk of suicidal behavior (HR and 95% CI 6.29, 4.48–8.83), and men in the second lowest category (2) had a four-fold higher risk of suicidal behavior (4.02, 2.89–5.60), compared with men in the highest intelligence category (5). In a similar manner, men in the lowest category of emotional control (1) had an almost seven-fold higher risk of suicidal behavior (6.64, 4.75–9.28), and men in the second lowest category (2) had a three-fold higher risk of suicidal behavior (3.46, 2.50–4.78), compared with men in the highest category (5).

When modeling intelligence and emotional control as continuous variables (parts b and d in Table 1, IQ and EC per unit decrease), suicidal behavior between 1973 and 2008 was associated with both lower intelligence (HR and 95% CI 1.54, 1.46–1.61 per unit decrease on the five-level scale) and lower emotional control (1.68, 1.60–1.76), after adjustment for childhood SES. This implies that one unit lower intelligence on the five-level scale was associated with a 54% higher risk of suicidal behavior and one unit lower emotional control was associated with a 68% higher suicidal risk. In the sensitivity analyses (both modeling intelligence and emotional control as continuous variables, per unit decrease; not shown in the table), when completed suicide was excluded from the dependent variable, the HR for intelligence (1.61, 1.52–1.70 per unit decrease) and the HR for emotional control (1.77, 1.67–1.88 per unit decrease) were only slightly higher; and excluding events of undetermined intent gave quite similar results to those of the main analysis (1.57, 1.47–1.67 for intelligence, and 1.73, 1.62–1.86 for emotional control, per unit decrease). Further, a crude analysis without controlling for childhood SES yielded very similar results (1.53, 1.46–1.60 for intelligence and 1.70, 1.61–1.78 for emotional control, per unit decrease), implying that childhood SES was not a confounder that would explain the associations.

**Fig. 1.**
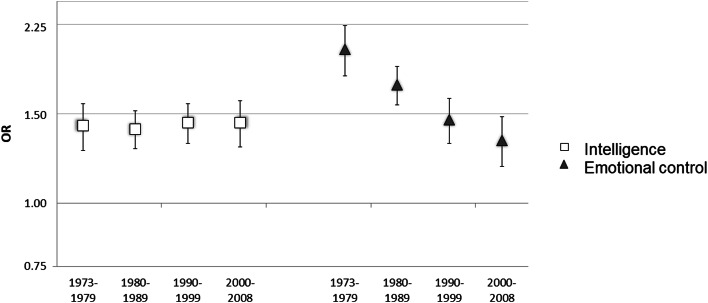
Associations between suicidal behavior and intelligence and emotional control, respectively, with adjustment for each other and for childhood SES. ORs with 95% CIs for suicidal behavior are given for a one step decrease on the five-level scales of intelligence and emotional control for the separate follow-up periods. The time periods end at about 10, 20, 30 and 40 years after conscription, respectively, and correspond with approximate age intervals of 22–28 years, 29–38 years, 39–48 years and 49–58 years.

When analyzed over the separate follow-up periods, the association between intelligence and suicidal behavior remained fairly stable over the life course, whereas the association between emotional control and suicidal behavior gradually weakened, see Table 1 (parts b and d; the continuous variables were used in these models). For example, one lower unit of intelligence was associated with a 65% higher risk of suicidal behavior during the first follow-up period (OR and 95% CI 1.65, 1.49–1.82) and a 53% higher risk of suicidal behavior during the last follow-up period (1.53, 1.39–1.69). On the other hand, one lower unit of emotional control was associated with a 117% higher risk of suicidal behavior during the first follow-up period (2.17, 1.95–2.40) but with only a 44% higher suicidal risk during the fourth and last follow-up period (1.44, 1.30–1.59). A significance test confirmed that the association with emotional control weakened over the follow-up period (*p* < 0.0001 for interaction with time). In the models with mutual adjustments for intelligence and emotional control, the associations were weakly to moderately attenuated but the difference between them over the total follow-up period remained, see Fig. 1. The exact estimates and 95% CIs are shown in online Supplementary Table S1.

## Discussion

In sum, our analyses showed that the association between low intelligence and suicidal behavior was fairly constant over the follow-up period to age 59 in this cohort of Swedish men, whereas the association between low emotional control and suicidal behavior weakened with increasing age. Mutual adjustment for intelligence and emotional control attenuated the associations somewhat but did not attenuate the difference between them. Adjustment for socioeconomic status during childhood had only marginal effects on the results.

### Findings in relation to previous research

Suicidal behavior has been linked to lower intelligence and higher neuroticism (which is similar to low emotional control) in some previous studies (Brezo *et al*., 2006; Osler *et al*., 2008; Gravseth *et al*., 2010; Wiktorsson *et al*., 2013), including a few separate studies of the same cohort of men as in the current study (Allebeck *et al*., 1988; Batty *et al*., 2010; Sörberg *et al*., 2013).

We are not aware of any previous studies focusing on changes in these associations over the life course, but a previous study using the same cohort showed that lower intelligence and a higher risk of hospitalization for major depression had a weakening association over the 38-year follow-up (Lager *et al*., 2017). Since depression is a well-known risk factor for suicide (Lesage *et al*., 1994), it is somewhat surprising that we found the association between higher intelligence and lower risk of suicidal behavior to remain stable over the life course. Nevertheless, risk factors for suicidal behavior are not identical to those for psychiatric disorders and their impact during the life span may differ (Gunnell and Lewis, 2005), which might explain the divergent findings.

### Possible explanations and implications

Although the associations of both intelligence and emotional control with suicidal behavior remained after adjustment, it is not necessarily the case that low intelligence and low emotional control are immediate causes of suicidal behavior. To some extent, these psychological differences between individuals may also predict suicidal behavior along indirect pathways. Several potential mechanisms underlying the associations of intelligence and neuroticism with suicidal behavior have been presented in previous research. For example, Gottfredson and Deary (2004) stress that intelligence may enhance individuals' care for their own health, since it represents learning, reasoning and problem-solving skills. Further, lower neuroticism (Gunthert *et al.*, 1999), and presumably also higher intelligence, are associated with more adaptive coping strategies, which might increase resilience in the face of stressful life events and thereby prevent suicidal behavior (Andersson *et al*., 2008; Wiktorsson *et al*., 2013). Also, it has been suggested that individuals with high neuroticism are more likely than others to experience stressful life events, and also gain less social support (Kendler *et al*., 2003; Deary *et al*., 2010), which may help to explain their increased risk of suicidal behavior.

One of the main findings of this study was that the association between intelligence and suicidal behavior remained constant over the entire follow-up period, while the association between emotional control and suicidal behavior was particularly strong in young adulthood and then weakened across the four periods. Largely, the two characteristics were associated with suicidal behavior independently of each other, as shown in the mutually adjusted models. Each, however, accounted for some of the other characteristic's association with suicidal behavior: for emotional control to a greater extent in the first follow-up periods, and for intelligence to an increasing extent in the later periods. Thus, mutual adjustment did not reduce the difference in time trends between intelligence and emotional control.

The gradual weakening of the association between emotional control and suicidal behavior may have some different potential explanations. One is that emotional control is more variable than intelligence from a life-course perspective. This idea is supported by previous research showing that there is a decrease in the population mean of neuroticism during late adolescence while intelligence seems to be more stable (Roberts *et al*., 2006). Similarly, previous research has shown that the presence of negative emotions, such as stress and anger, steeply decline from the early 20s, and that older adults are less emotionally reactive to stressors and less engaged in destructive conflict strategies than younger adults (Stone *et al*., 2010; Mather, 2012). Another potential explanation for why the association weakens is that stressful circumstances or life events during the years leading up to the conscription examination, e.g. in school (Lager *et al*., 2016), could both have had a negative impact on emotional control, as rated in the psychologists' interviews at conscription, and increased the incidence of suicidal behaviors for a time following the conscription examination. This would be consistent with findings showing that some of the fluctuations in neuroticism over time seems to be associated with life events (Ormel *et al*., 2012). In principle, however, the conscription interview was designed to measure a personality trait, not an emotional state.

Our results highlight the possibility that low intelligence and – less surprisingly – poor emotional control influence the risk of suicide. The importance of this for suicide-preventive work may be worth investigating further. Other studies have discussed how low intelligence and poor emotional control also increase the likelihood of ending up in difficult circumstances, e.g. with low education or problematic social relations (Roberts *et al*., 2007; Deary *et al*., 2010). In accordance with this, studies in which we ourselves were involved have suggested that low socioeconomic status, and also risky behaviors, in young adulthood help to explain the relationships between intelligence, emotional control and health outcomes later in life (Sörberg *et al*., 2013; Potijk *et al*., 2016). In particular, our results may justify paying increased attention to the evidence that neuroticism and poor emotional control may contribute to high but eventually diminishing rates of harmful behaviors, including suicide, in young adult men. Access to treatment for the young may have to be given priority, since there is some evidence that neurotic problems can be reduced, e.g. with the help of cognitive behavioral therapy (CBT) and similar forms of psychotherapy (Jorm, 1989; Tang *et al*., 2009).

On the other hand, one possible reason for the long-term association between intelligence and suicidal behavior is that intelligence is robustly associated with attained education and socioeconomic position in adulthood (Plomin and Deary, 2015). These factors have been found to explain substantial proportions of the association between intelligence in youth and suicidal behavior in adulthood (Sörberg *et al*., 2013; Sörberg Wallin *et al*., 2018*a*). They may be relevant to prevention at the societal level, not least because socioeconomic factors contribute considerably to suicide risk at the population level (Li *et al*., 2011). Of note is that this pathway might be more important for suicide prevention in men than in women; in women, low intelligence seems to be associated with suicide attempt (Sörberg Wallin *et al*., 2018*b*) but not death from suicide (Andersson *et al*., 2008; Calvin *et al*., 2017; Sörberg Wallin *et al*., 2018*a*). The fact that gender differences are observed in these relationships further supports the hypothesis that social and socioeconomic factors are involved as mediators or moderators in the associations.

### Strengths and limitations

There are several strengths to this study. The study population is strongly representative of the Swedish population of men who were born in 1950 and 1951, since nearly all men participated in compulsory conscription at that time. The exposure variables were measured using standardized test batteries and semi-structured interviews following manuals, and the outcome variables are based on national records with high reliability (Lilieblad and Ståhlberg, 1977; Ross, 1988). Further, the 38-year follow-up period enabled us to follow the men in the cohort over a substantial part of their lives.

There are, however, two aspects of this study that limits its generalizability. First, we cannot generalize the results to women since only men were obliged to participate in conscription. Second, the results cannot be directly extrapolated to other settings partly because suicidal behavior differs between cultures (Nock *et al*., 2008). Therefore, generalizations to other populations should be made with these limitations in mind. Further, our outcome variable, suicidal behavior, includes both completed and attempted suicide. This merged variable has been used in previous studies of the same cohort (Åberg *et al*., 2014), but since suicide attempt is far more frequent than completed suicide (Leon *et al*., 1990), and behavior associated with completed suicide may differ from behavior associated with suicide attempt, the merger might seem debatable. However, because our data include only severe suicide attempts that have led to hospitalization, it can be assumed that attempted and completed suicides are quite similar in the present study. In our analyses, the estimates were only slightly higher when completed suicide was excluded from the dependent variable. Lastly, intelligence and emotional control were measured at one point in time only. While intelligence is quite stable over the life course (Gow *et al*., 2011), personality aspects such as emotional control are more variable. Data with repeated measures of personality aspects are needed to directly investigate how changes in these measures might explain changes in their relationship with suicidal behavior.

## Conclusions

In line with previous research, this study confirms that low intelligence and low emotional control are both associated with a higher risk of suicidal behavior among men. Further, this study adds to previous research by showing that the association between intelligence and suicidal behavior seems constant over the life course, whereas the association between emotional control and suicidal behavior may become considerably weaker over time. The particularly high suicide risk of young men with poor emotional control may motivate targeted prevention efforts.

## Supplementary material

For supplementary material accompanying this paper visit https://doi.org/10.1017/S0033291719002423. click here to view supplementary material
