# Supplementary material for: How intelligence and emotional control are related to suicidal behavior across the life course – A register-based study with 38-year follow-up
Source: Psychol Med. 2019 Oct 9;50(13):2265–71. doi: 10.1017/S0033291719002423 (PMC7557158; doi:10.1017/S0033291719002423)
Supplement: Supplementary file 1 [file S0033291719002423sup001.docx]

Supplementary material to *How intelligence and emotional control are related to suicidal behavior across the life course* by N. Hansson Bittár, D. Falkstedt and A. Sörberg Wallin.

Table S1. Associations between intelligence (IQ) and emotional control (EC) and suicidal behavior in the four separate time periods, in odds ratios (ORs), with 95% confidence intervals (CIs) per one unit decrease in IQ/EC, in models adjusting for 1) childhood SES, for comparison, and 2) childhood SES and IQ or EC (mutual adjustment).

|  | **Model 1: adjusted for childhood SES** | | **Model 2: adjusted for childhood SES and IQ/EC** | |
| --- | --- | --- | --- | --- |
|  | **OR** | **95% CI** | **OR** | **95% CI** |
| b. IQ, per unit decrease |  |  |  |  |
| 1973–1979 | 1.65 | 1.49-1-82 | 1.42 | 1.29-1.57 |
| 1980–1989 | 1.57 | 1.45-1.70 | 1.40 | 1.29-1.52 |
| 1990–1999 | 1.56 | 1.43-1.70 | 1.44 | 1.32-1.57 |
| 2000–2008 | 1.53 | 1.39-1.69 | 1.44 | 1.30-1.59 |
| d. EC, per unit decrease |  |  |  |  |
| 1973–1979 | 2.17 | 1.95-2.40 | 2.01 | 1.80-2.24 |
| 1980–1989 | 1.84 | 1.69-2.00 | 1.71 | 1.56-1.86 |
| 1990–1999 | 1.58 | 1.45-1.73 | 1.46 | 1.33-1.61 |
| 2000–2008 | 1.44 | 1.30-1.59 | 1.33 | 1.20-1.48 |
